# Fecal microbiota and diets of muskox female adults and calves

**DOI:** 10.1002/ece3.8879

**Published:** 2022-05-03

**Authors:** Ji‐Yeon Cheon, Hyunjun Cho, Mincheol Kim, Hyun Je Park, Tae‐Yoon S. Park, Won Young Lee

**Affiliations:** ^1^ 123591 Division of Life Sciences Korea Polar Research Institute Incheon Korea; ^2^ 34973 Department of Environmental Science and Ecological Engineering Korea University Seoul Korea; ^3^ 34961 Department of Marine Bioscience Gangneung‐Wonju National University Gangneung Korea; ^4^ 123591 Division of Earth Sciences Korea Polar Research Institute Incheon Korea; ^5^ 123591 Polar Science University of Science & Technology Daejeon Korea

**Keywords:** diet analysis, gut microbiome, high Arctic, large herbivore, *Ovibos moschatu*

## Abstract

In mammals, the gut microbiome is vertically transmitted during maternal lactation at birth. In this study, we investigated the gut microbiome and diets of muskox, a large herbivore inhabiting in the high Arctic. We compared the microbiota composition using bacterial 16S rRNA gene sequencing and diets using stable isotope analysis of muskox feces of six female adults and four calves on Ella Island, East Greenland. Firmicutes were the most abundant bacterial phylum in both the adults and calves, comprising 94.36% and 94.03%, respectively. Significant differences were observed in the relative abundance of the two Firmicutes families. The adults were primarily dominated by Ruminococcaceae (73.90%), and the calves were dominated by both Ruminococcaceae (56.25%) and Lachnospiraceae (24.00%). Stable isotope analysis of the feces in the study area revealed that both adults and calves had similar ranges of ^13^C and ^15^N, likely derived from the dominant diet plants. Despite their similar diets, the different gut microbiome compositions in muskox adults and calves indicate that the gut microbiome of the calves may not be fully colonized to the extent of that of the adults.

## Graphical abstract

We present fecal microbiota and diet compositions of muskox female adults and calves in northeast Greenland. By 16S sequencing and stable isotope analysis, we found that the mother and calf have different microbial communities despite the similar diet. We discuss that the gut microbiome of the calves may not be fully colonized yet as much as one of the adults.

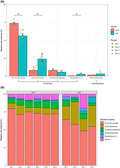

## 1 INTRODUCTION

Animals are associated with a diverse gut microbiome, which affects the health, immunity, and metabolites of the host (Kinross et al., 2011). The gut microbiome composition may change with the development, diet, and surrounding environment of the host (Eckburg et al., 2005; Xu & Knight, 2015). Therefore, the gut microbiome may provide an important insight into the ecology of host animals and may be related to pathogens that can cause zoonotic diseases (Andersen‐Ranberg et al., 2018). To date, the gut microbiome research has been primarily focused on humans or captive animals; however, the gut microbiome and its related functions in wild animals remain poorly understood (Davidson et al., 2020).

In mammals, the gut microbiome can be vertically transmitted from birth through parental care during the lactation phase and birth process by directly delivering maternal materials to offspring. Consequently, it has a significant impact on gut microbiome formation in the early growth stage of the offspring (Chu et al., 2016; Wang et al., 2020). In a mouse model study, most microbiota genera have been reported to be vertically transmitted over generations (Moeller et al., 2018). In addition to the vertical transmission, diet is a major factor that facilitates gut microbiome formation. The microbiota can also be indirectly affected by the diet acquisition of newborns in different food conditions provided by nursing mothers (Frese et al., 2015). The composition of a starter diet can vary among families, and therefore, it can shape microbial structure and functions in digestion.

In this study, we investigated feces collected from female adults and calves of the muskox (*Ovibos moschatus*) during the summer in high Arctic environments. The muskox is a large herbivorous mammal that inhabits in the Arctic environment (Cuyler et al., 2020). Diets are influenced by local plants and seasonal availability. In Jameson Land, east Greenland, the dominant diets are willows (*Salix* spp.) in summer and graminoids (*Carex* and *Eriophorum*) in winter (Thing et al., 1987). In the Zachkenberg Valley, east Greenland, muskoxen were observed to forage in grasslands (dominated by graminoids (Cyperaceae, Juncaceae, and Poaceae), wideleaf polargrass (*Arctagrostis latifolia*), alpine foxtail (*Alopecurus magellanicus*) and cottongrass), fens and *Salix* snowbeds in summer while willows (*Salix* spp.), horsetail (*Equisetum variegatum*), and dwarf shrubs (*Dryas* spp.) (Kristensen et al., 2011; Schmidt et al., 2018). Calves are typically born between April and May (Schmidt et al., 2020) and commence grazing one week after birth while remaining closely attached to their mothers. Calves wean completely after one year (Adamczewski et al., 1994). The gut microbiomes and diets of female adults and calves were compared via bacterial 16S rRNA gene sequencing and stable isotope analysis. This study addressed the following questions: (a) whether muskoxen have different gut microbiomes with age (female adults vs. calves) and (b) whether the two age groups have similar diets.

## 2 MATERIALS AND METHODS

### 2.1 Study site and fecal sample collection

**FIGURE 1:**
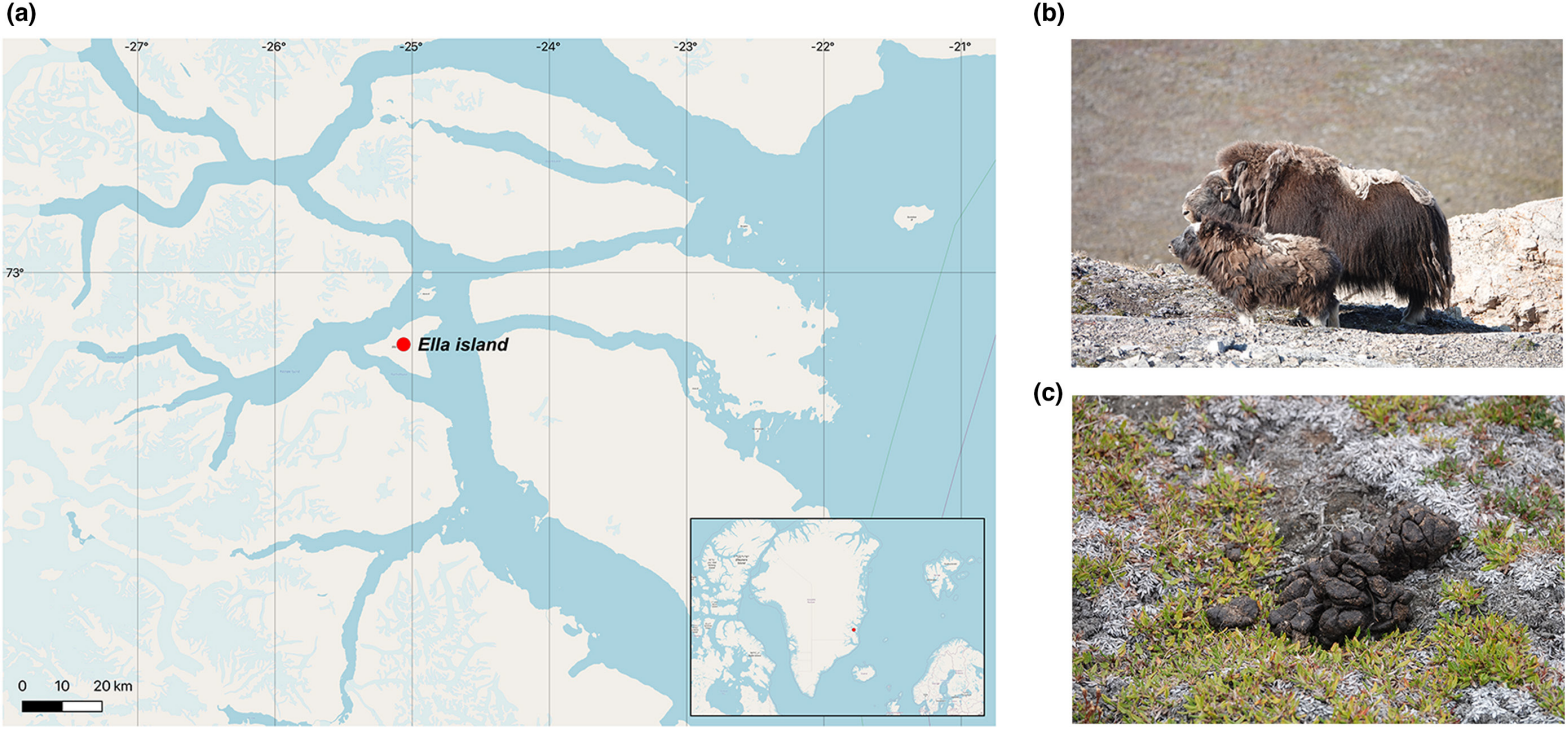
(a) The location of the study site, Ella Island in East Greenland (72°50′N, 25°00′W); (b) muskox female adult and calf in August 2019; (c) muskox fecal sample

The samples were collected during August 2019 from Ella Island (72°50′N, 25°00′W), which is located in East Greenland (Figure 1). Ella Island presents a dry environment, with low temperatures not exceeding 10℃, even during summer (Kottek et al., 2006). In east Greenland, the dominant vegetation comprises willows, grasses, and sedges, particularly dwarf shrubs (Arndal et al., 2009; Kristensen et al., 2011; Schmidt et al., 2018).

Ten muskox fresh fecal samples were collected, with six from muskox female adults and four from calves (four pairs of mother and calf and two females with no calf). Eight samples comprised four pairs of mother and calves. When the animals were observed, a pair of researchers had waited for defecation in a few hundred meters away. Because we did not mark the individuals, we could not make sure that all individuals were different from each other. Despite the lack of individual marking, the feces of the female adult and calf were distinguishable based on the defecating locations and the amount of feces. To avoid soil contamination, we used sterile gloves and spoons to collect the fecal materials that were not touched on the ground (Yang et al., 2016). All samples were kept in an ethanol solution (99%) until DNA extraction (September 2019). As candidate prey sources, the leaves, stems, and fruits of eight plant species were collected from the ground where the muskox foraged.

### 2.2 DNA extraction, amplification, and sequencing

Fecal DNA was individually extracted from subsamples at more than 3 different regions of the original sample, using the QIAGEN QIAamp Fast Stool Mini Kit according to the manufacturer's instructions. DNA was amplified by targeting the V3‐V4 region of the bacterial 16S rRNA gene using primers, 341F (5′‐CCTAGGGGNGGCWGCAG‐3′) and 805R (5′‐GACTACHVGGGTATCTAATCC‐3′) (Klindworth et al., 2013), and amplification was performed using the following protocol: one denaturation step at 94°C for 3 min, five cycles of denaturation at 94°C for 15 s and extension at 65°C for 60 s, 20 cycles of denaturation at 94°C for 1 min, annealing at 55°C for 20 s and extension at 72°C for 30 s, and a final extension at 72°C for 5 min. Sequencing library construction and amplicon sequencing were performed at Macrogen (Seoul, South Korea) using a 2 × 300 bp Illumina MiSeq sequencing system (Illumina, USA).

### 2.3 Bioinformatic analyses

The adapters and primers from the raw sequence reads were trimmed using Cutadapt v2.10 (Martin, 2011). The bioinformatics pipeline was run using DADA2 v1.16 (Callahan et al., 2016) to infer amplicon sequence variants (ASVs) with single‐nucleotide resolution. For quality trimming, a more relaxed filtering option was applied to the reverse reads as maxEE = c (2, 5), and the low‐quality sequence tails were removed from the forward and reverse reads with truncLen = c (270, 210). Bacterial taxonomy was assigned to representative ASV sequences using the DADA2 implementation of the RDP‐naive Bayesian classifier based on the EzBiocloud database (Yoon et al., 2017). Sequences matched to the Eukaryota, Archaea, or Cyanobacteria were removed from the data set. Sequences are available in the NCBI Sequence Read Archive (SRA) database under the accession number PRJNA753257.

### 2.4 Stable isotope analysis

For stable isotope analysis, 1‐mg muskox feces was homogenized. Each sample was freeze‐dried and prepared using a stable isotope ratio mass spectrometer system (IsoPrime 100; Cheadle, UK) with a vario MICRO cube elemental analyzer (Elementar, Hanau, Germany). Purified CO_2_ and N_2_ were used as the sample analysis gas and the isotopic reference gases, respectively. The GC column resolves CO_2_ from N_2_, and the reduction column filled with copper wires reduces N_2_. All results are reported with delta notation, in parts per thousand (‰) relative to the PDB standard. Each plant sample was analyzed six times during this analysis.

$$\delta^{13}\text{C and}\;\delta^{15}N=\left(R_{\text{sample}}/R_{\text{standard}}‐1\right)\times 1000\;\left(‱\right)$$

The international reference materials of sucrose (ANU C12H22O11; NIST, Gaithersburg, MD, USA) for δ13C and ammonium sulfate ([NH4]2SO4; NIST) for δ15N were analyzed to calibrate the reference gases and the internal standard (acetanilide; Thermo Scientific). The analytical precision was based on 10 replicate measurements of acetanilide and was within 0.12‰ and 0.20‰ for δ^13^C and δ^15^N, respectively.

### 2.5 Statistical analysis

To correct the differences in the number of reads, all samples were subsampled to the level of the smallest number of reads found in the samples. Bray–Curtis dissimilarities between all sample pairs were calculated using a Hellinger‐transformed ASVs abundance matrix and visualized using nonmetric multidimensional scaling (NMDS). The permutational multivariate analysis of variance nonparametric test (PERMANOVA) was used to test for the differences in bacterial community structure between the two groups of the muskox using PRIMER 6 and PERMANOVA+ (Clarke & Tobutt, 2003).

The age group (fixed with two levels comprising adult and calf) was considered as a fixed component, and *p*‐values were obtained using 999 permutations. We used the three indices to estimate the bacterial diversity and compared the diversity values between the two groups of the muskox by age using the *t*‐test and Fligner–Killeen test. Rarefaction curves and the stable isotope analysis results were generated using R packages (version 4.0.5, http://www.R‐project.org). Bacterial abundances were compared between the age groups (adults vs. calves), and the four pairs of adults and calves were additionally tested.

Bacterial functional abundances were inferred using PICRUSt2 v.2.3.0b (Douglas et al., 2020), and the predicted microbial functions (KEGG orthologs) were visualized with a principal coordinates analysis (PCoA) plot.

## 3 RESULTS

**FIGURE 2:**
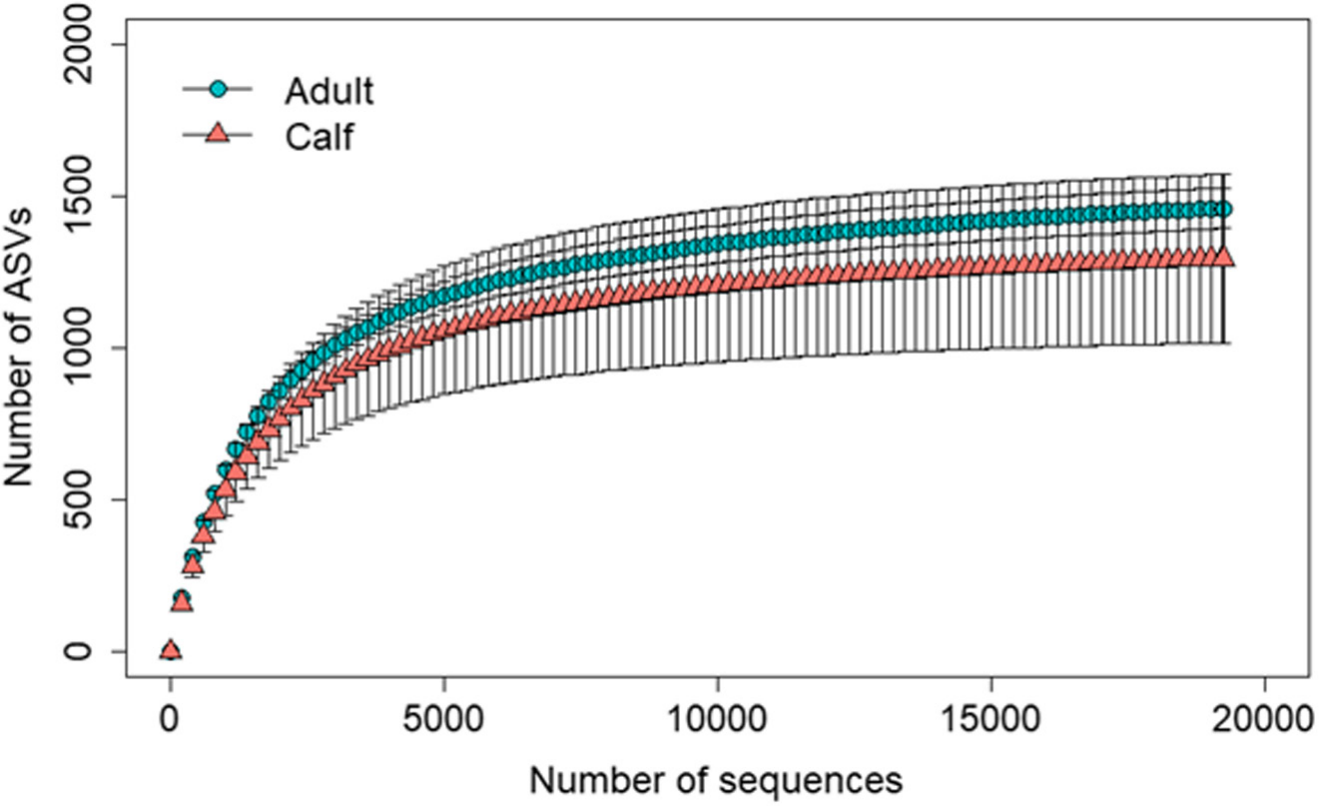
Rarefaction curves of fecal bacterial communities between muskox adult and calf muskox groups

We obtained a total of 335,970 high‐quality bacterial 16S rRNA gene sequences from all muskox fecal samples, ranging from 19,222 to 42,610 sequences per sample. The rarefaction curves showed that it almost attained the saturation plateau, indicating that the sample coverages were sufficiently large enough to estimate the ASV richness (Figure 2).

**FIGURE 3:**
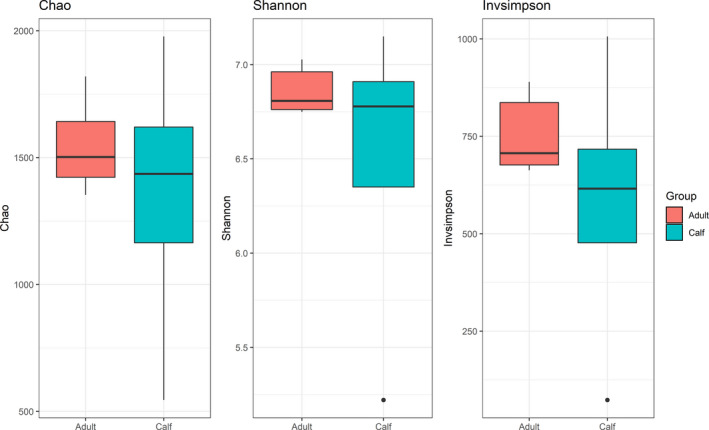
Bacterial alpha diversity (3 indices; Chao, Shannon, and Invsimpson) in muskox adults (*n* = 6) and calves (*n* = 4) presented on box‐whisker plots. No significant mean differences are detected between adults and calves (*t*‐tests; Chao, *p* = .56; Shannon, *p* = .45; Invsimpson, *p* = .43)

Adult bacterial diversity was not higher than those of calves in the Chao, Shannon, and Invsimpson indices, respectively (Chao index; adults = 1543.9, calves = 1348.7, Shannon index; adults = 6.86, calves = 6.48, Invsimpson; adults = 752, calves = 577.8, on average); the differences were not statistically significant (*t*‐tests; Chao, *p* = .56; Shannon, *p* = .45; Invsimpson, *p* = .43) (Figure 3). Instead, there were differences in the group variances of the three diversity indices between adults and calves (Fligner–Killeen tests; Chao, *p* = .03; Shannon, *p* < .01; Invsimpson, *p* = .02; Figure 3).

**FIGURE 4:**
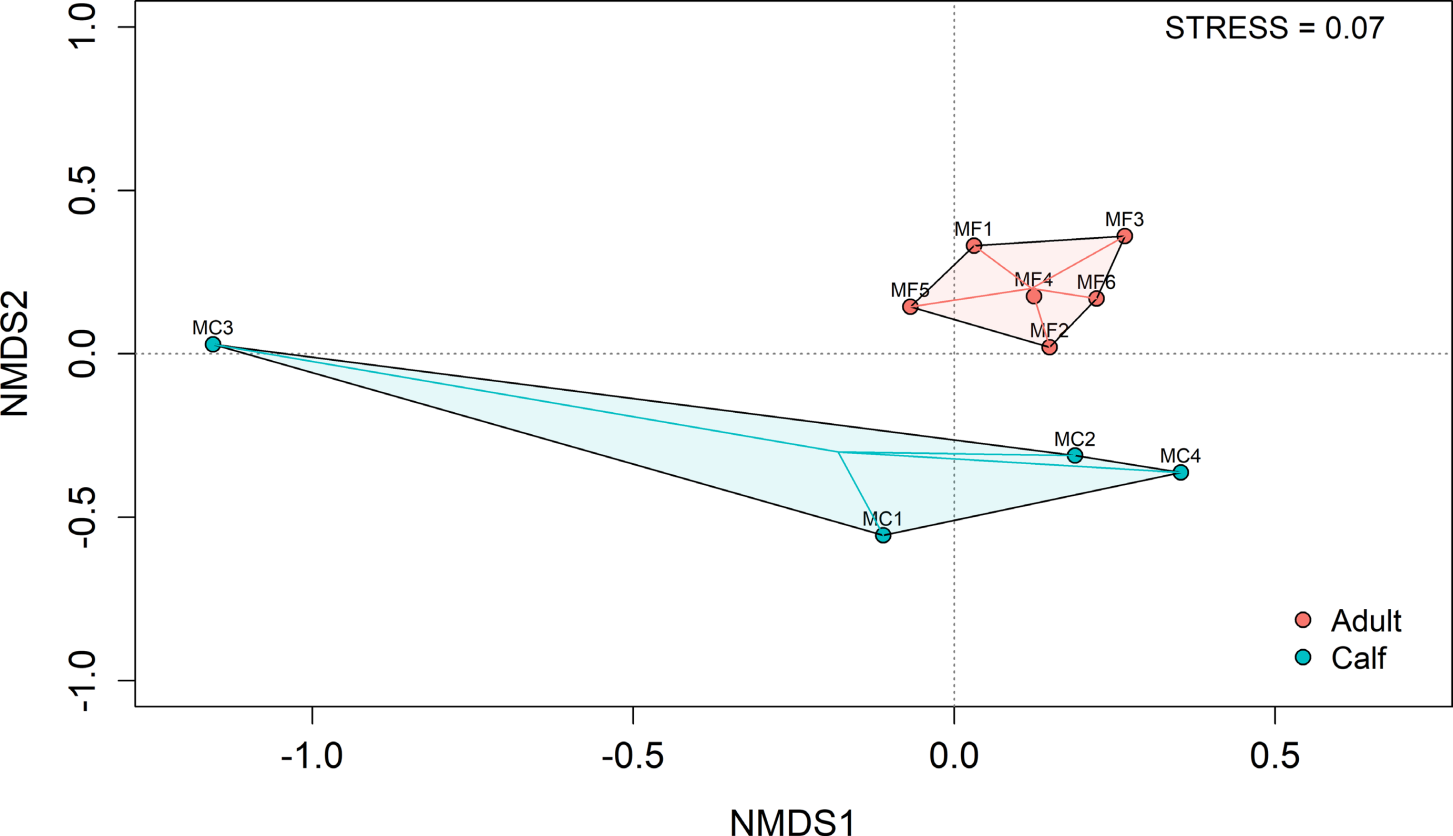
Nonmetric multidimensional scaling (NMDS) plot of muskox fecal bacterial communities using Bray–Curtis dissimilarity measures. All the points within each group are connected to the group centroid

The NMDS plot showed bacterial community differences between muskox adults and calves (PERMANOVA, pseudo‐*F* = 1.69, *p* < .01) (Figure 4).

**FIGURE 5:**
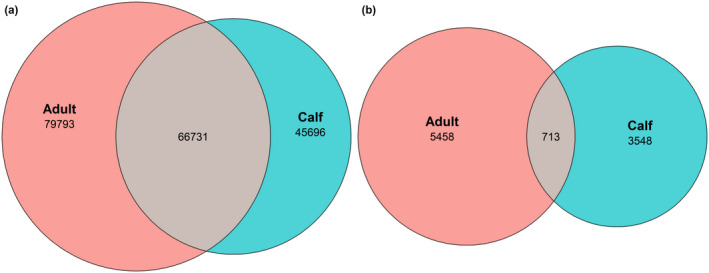
Venn diagram showing (a) the total sequence reads and (b) the number of unique and shared bacterial ASVs in adult and calf groups

We attempted to identify bacterial ASVs, which were shared between different groups of the muskox. A total of 34.72% (66731/125489) of the total sequence reads were shared, and 7.33% (713/9719) of the total ASVs were shared between adults and calves (Figure 5). Ruminococcaceae (adults: 77.96%, calves: 60.64%) and Lachnospiraceae (adults: 8.79%, calves: 26.69%) were abundant in the shared ASVs.

**FIGURE 6:**
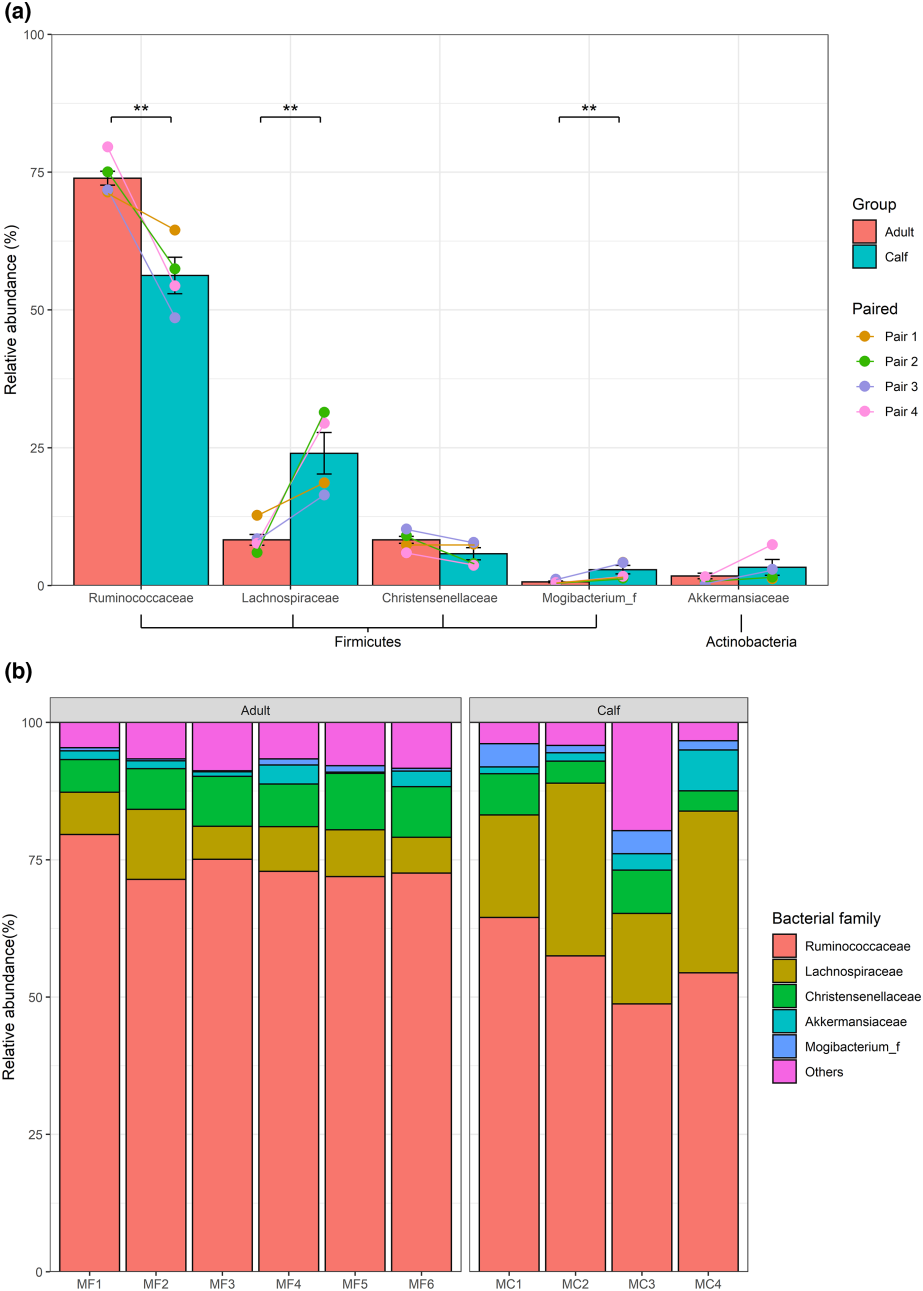
(a) Relative abundance of dominant bacteria family of the total number of ASVs in muskox adults and calves. Bar plot shows the relative abundance of adults (*n* = 6) and calves (*n* = 4), *t*‐tests; asterisks indicate the significance of the statistical test of differences between adults and calves (asterisks *means *p* < .05, **means *p* < .01, ***means *p* < .001). Four different color dots and lines show the relative abundance of adults (*n* = 4) and calves (*n* = 4) with paired samples (Pair 1–4). (b) Distribution of bacterial families across all fecal samples from muskox individuals

At the phylum level, the muskox gut microbiome was dominated by Firmicutes (on average, 94.36% and 94.03%) and Verrucomicrobia (1.77% and 3.31%, respectively) in both adults and calves. These two phyla accounted for 91.16% of the total sequences from all the samples. At the family level, we found that five families were dominant. These were Ruminococcaceae (73.90% and 56.25%), Lachnospiraceae (8.27% and 24.00%), Christensenellaceae (8.28% and 5.76%), Mogibacterium_f (0.65% and 2.86%), and Akkermansiaceae (1.72% and 3.30%) (Figure 6a,b). The relative abundance of the three microbial families differed significantly between muskox adults and calves (Ruminococcaceae, *p* < .01; Lachnospiraceae, *p* < .01; Mogibacterium_f, *p* < .01; Mann–Whitney tests; Figure 6a). Although the four pairs of adults and calves did not exhibit significant differences (paired Mann–Whitney tests; indicated by colored lines in Figure 6a), Ruminococcaceae and Christensenellaceae exhibited consistent increases, whereas Lachnospiraceae, Mogibacterium_f, and Akkermansiaceae exhibited consistent decreases in all pairs.

**FIGURE 7:**
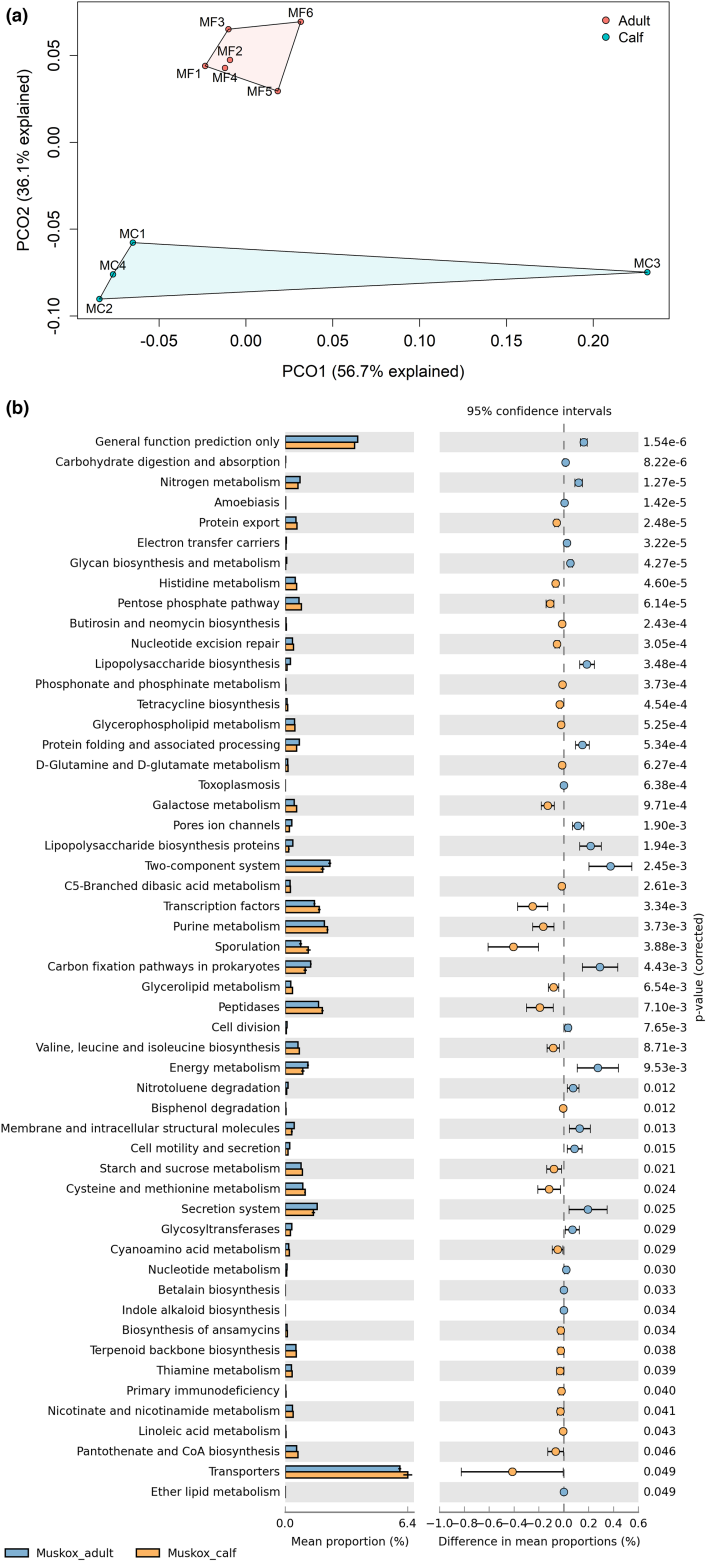
(a) Principal coordinates analysis (PCoA) plot of PICRUSt2‐predicted functions of muskox fecal microbiota using Bray–Curtis dissimilarity measures (adults: MF1–6, *n* = 6, calves: MC1–4, *n* = 4). (b) PICRUSt2‐predicted microbial functions with significant differences between the muskox adults and calves at level 3 KEGG functional categories

**FIGURE 8:**
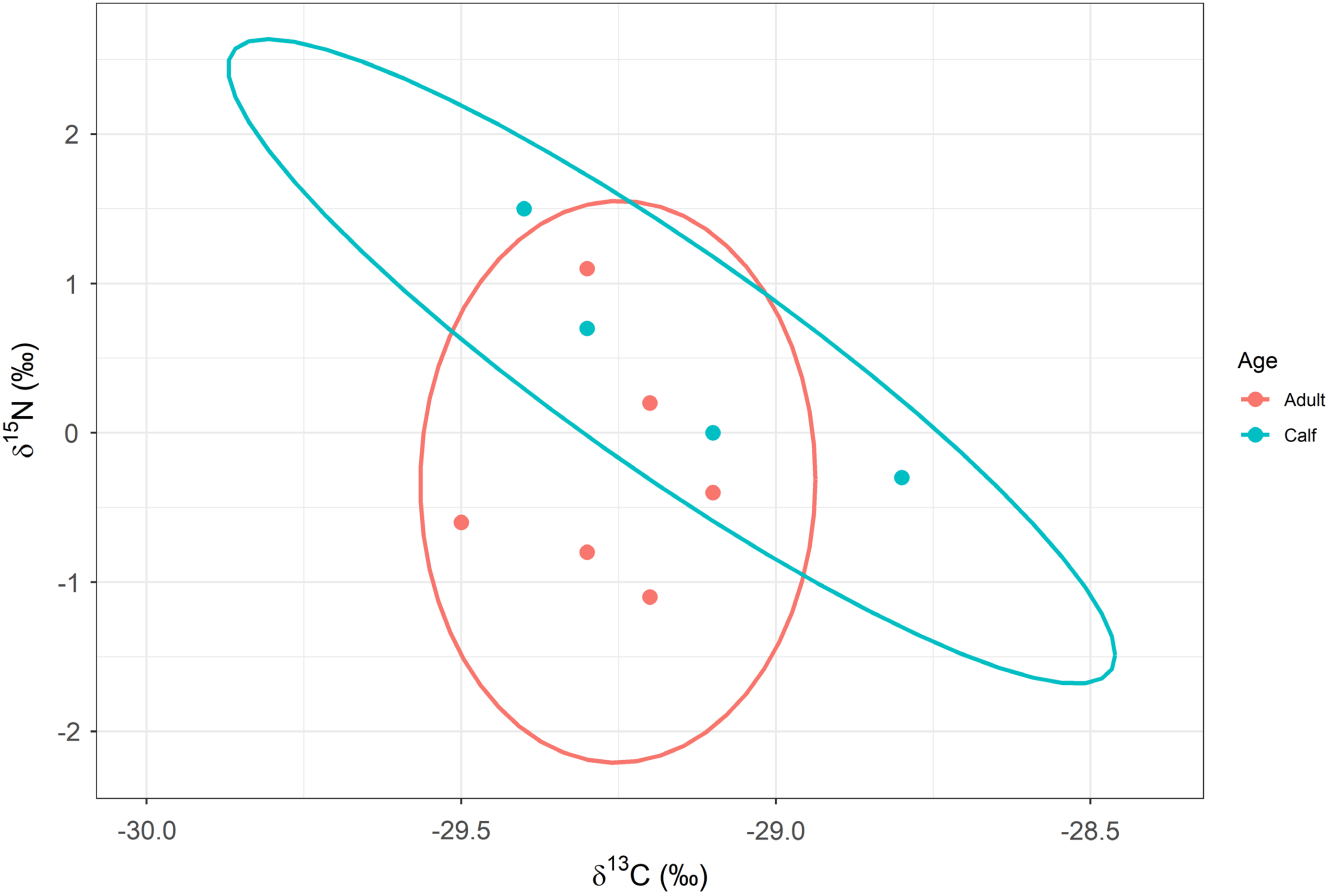
Stable isotopic niche for muskox adults (*n* = 6), calves (*n* = 4). Ellipses represent 90% confidence interval in each muskox group

The microbial functional structure differed between muskox adults and calves (PERMANOVA, pseudo‐*F* = 3.63, *p* = .01) (Figure 7). Among the predicted functions at KEGG level 3, 54 functions were significantly different between muskox adults and calves were provided in Figure 8. Transporters was the dominant pathway in both muskox adults and calves (5.97% vs. 6.39%, *p* < .05). The calves had higher purine metabolism (2.21% vs. 2.05%, *p* < .01) and peptidases function (1.94% vs. 1.75%, *p* < .01) while the adults had higher secretion system (1.67% vs. 1.47%, *p* < .05) and energy metabolism function (1.19% vs. 0.92%, *p* < .01).

Stable isotopic niches for muskox adults (*n* = 6) and calves (*n* = 4) are presented in Figure 8. Muskox adults and calves had similar values in δ^13^C and δ^15^N (PERMANOVA, *p* = .413; adults, δ^13^C = –29.15 ± 0.13, δ^15^N = –0.26 ± 0.32; calves, δ^13^C = –29.15 ± 0.13, δ^15^N = 0.47 ± 0.4). Two groups of ellipses were overlapped in 47.6% of the area of adults and 48.1% of the area of calves.

## 4 DISCUSSION

The results showed that the microbiomes of muskox adults and calves have similar levels of alpha diversity at the phylum and family levels, although the calves exhibited higher variance values. The adults and calves had different bacterial communities, and the calves exhibited a more diverse composition within the group compared with the adults. The dietary analysis indicated that the adults and calves had common diets. The different bacterial communities between female adults and calves with similar diets, suggest that the gut microbiome in the calf group is still developing and not fully colonized despite their dietary similarities. After birth, calves receive the gut microbiome from mothers and begin to form their own independent gut microbiomes (Barko et al., 2018). Muskox calves graze from three to six weeks after birth and follow their mothers to select dietary plants (Church, 1969). During the study period, it was also observed that the females and calves foraged together. The diet analysis results confirmed that the adults and calves foraged on the same plants. When considering the breeding cycle (birth around March or April) (Adamczewski et al., 1994), the calves in this study are assumed to be three or four months old. Therefore, we show that the calf gut microbiome reaches the developing stage by August.

The dominant phylum in the fecal samples was Firmicutes. At the family level, Ruminococcaceae and Lachnospiraceae, which belong to the class Clostridia and phylum Firmicutes, were dominant, occupying more than 80% of the total abundance. Ruminococcaceae and Lachnospiraceae were reported to encode carbohydrate‐active enzymes for glycoside hydrolases and carbohydrate esterases in herbivores (Wang et al., 2016). Ruminococcaceae is also known to affect the secondary metabolite synthesis and is involved in host immunity, such as antibiotic biosynthesis (Gosalbes et al., 2011) by producing short‐chain fatty acids (SCFAs) for lipid metabolism and digestion (Morrison & Preston, 2016) and by detoxifying the plant secondary metabolites (Kohl et al., 2014). Lachnospiraceae has been reported to produce SCFAs for metabolism (Hao et al., 2017; Vacca et al., 2020) and digest lactose by converting lactate into butyrate (Meehan & Beiko, 2014). In this study, adults had more Ruminococcaceae and fewer Lachnospriaceae than calves. Such differences could be related to the microbial functions for host digestion and metabolism, depending on their need. We infer that the differences could result in differential needs for digestion between the adults and calves because calves were still relying on the milk during the sampling period.

The predicted microbial functions indicates that transporters and metabolisms were dominant. We found pathways to help the digestion of dietary fibers for carbohydrates (carbohydrate metabolism, adults: 0.11%, calves: 0.13%) and lipids (fatty acid metabolism, adults: 0.41%, calves: 0.33%; glyoxylate and dicarboxylate metabolism, adults: 0.76%, calves: 0.65%; fatty acid biosynthesis, adults: 0.60%, calves: 0.57%).

The microbial results presented here are consistent with those of previous studies on large herbivores. In the previous muskox studies, Firmicutes was the most dominant phylum (74–83%), and Ruminococcaceae and/or Lachnospiraceae were the most dominant families (from Norway and northeast Greenland in Andersen‐Ranberg et al., 2018; from Norway in Salgado‐Flores et al., 2016; from Canada in Bird et al., 2019). In Svalbard and Norwegian reindeers, the phylum Firmicutes and the families Ruminococcaceae and Lachnospiraceae were also abundant (Sundset et al., 2007; Zielińska et al., 2016). Based on previous studies and our PICRUSt2 results, we suggest that Ruminococcaceae and Lachnospiraceae could promote cellulose metabolism in herbivores.

From the stable isotope analysis, we found that adults and calves shared similar diets. In previous studies, *Salix* spp. was found to be the main food source of muskoxen, particularly during the summer (Gustine et al., 2014; Thing et al., 1987), demonstrating a relatively high digestibility for muskoxen (Staaland & Olesen, 1992).

The present findings may provide ecological information for understanding the host and microbial interactions and provide insights into the microbial functions for digestion in herbivores. In future studies, it will be interesting to analyze the detailed microbial functions related to their digestion and immune functions in adults and calves.

## CONFLICT OF INTEREST

The authors have declared that no competing interests exist.

## AUTHOR CONTRIBUTIONS

**Ji‐Yeon Cheon:** Formal analysis (equal); Writing – original draft (lead); Writing – review & editing (equal). **Hyunjun Cho:** Data curation (equal); Formal analysis (equal); Writing – review & editing (equal). **Mincheol Kim:** Data curation (equal); Formal analysis (equal); Writing – review & editing (equal). **Hyun Je Park:** Formal analysis (equal); Writing – review & editing (equal). **Tae‐Yoon S. Park:** Funding acquisition (lead); Supervision (equal); Writing – review & editing (equal). **Won Young Lee:** Conceptualization (lead); Formal analysis (equal); Investigation (lead); Supervision (equal); Writing – original draft (lead); Writing – review & editing (lead).

## Data Availability

The data that support the findings of this study are openly available in NCBI Sequence Read Archive (SRA) database under accession number “PRJNA753257” (https://www.ncbi.nlm.nih.gov/sra/?term=PRJNA753257).
