# Corrigendum

**DOI:** 10.1002/ece3.8969

**Published:** 2022-05-24

In the recent article by Cheon et al. (2022), the correct grant number of “Korea Polar Research Institute” should be "PE22060" in the Funding information section.

The authors apologize for the error.
